# Ethylene and Jasmonates Signaling Network Mediating Secondary Metabolites under Abiotic Stress

**DOI:** 10.3390/ijms24065990

**Published:** 2023-03-22

**Authors:** Marina Pérez-Llorca, Stephan Pollmann, Maren Müller

**Affiliations:** 1 Department of Biology, Health and the Environment, Faculty of Pharmacy and Food Sciences, University of Barcelona, 08028 Barcelona, Spain; 2 Centro de Biotecnología y Genómica de Plantas, Instituto Nacional de Investigación y Tecnología Agraria y Alimentación (INIA/CSIC), Universidad Politécnica de Madrid (UPM), Campus de Montegancedo, Pozuelo de Alarcón, 28223 Madrid, Spain; 3 Departamento de Biotecnología-Biología Vegetal, Escuela Técnica Superior de Ingeniería Agronómica, Ali-Mentaria y de Biosistemas, Universidad Politécnica de Madrid (UPM), 28040 Madrid, Spain; 4 Department of Evolutionary Biology, Ecology and Environmental Sciences, Faculty of Biology, University of Barcelona, 08028 Barcelona, Spain

**Keywords:** ethylene, jasmonates, abiotic stress, secondary metabolism, signaling

## Abstract

Plants are sessile organisms that face environmental threats throughout their life cycle, but increasing global warming poses an even more existential threat. Despite these unfavorable circumstances, plants try to adapt by developing a variety of strategies coordinated by plant hormones, resulting in a stress-specific phenotype. In this context, ethylene and jasmonates (JAs) present a fascinating case of synergism and antagonism. Here, Ethylene Insensitive 3/Ethylene Insensitive-Like Protein1 (EIN3/EIL1) and Jasmonate-Zim Domain (JAZs)-MYC2 of the ethylene and JAs signaling pathways, respectively, appear to act as nodes connecting multiple networks to regulate stress responses, including secondary metabolites. Secondary metabolites are multifunctional organic compounds that play crucial roles in stress acclimation of plants. Plants that exhibit high plasticity in their secondary metabolism, which allows them to generate near-infinite chemical diversity through structural and chemical modifications, are likely to have a selective and adaptive advantage, especially in the face of climate change challenges. In contrast, domestication of crop plants has resulted in change or even loss in diversity of phytochemicals, making them significantly more vulnerable to environmental stresses over time. For this reason, there is a need to advance our understanding of the underlying mechanisms by which plant hormones and secondary metabolites respond to abiotic stress. This knowledge may help to improve the adaptability and resilience of plants to changing climatic conditions without compromising yield and productivity. Our aim in this review was to provide a detailed overview of abiotic stress responses mediated by ethylene and JAs and their impact on secondary metabolites.

## 1. Introduction

The effects of climate change affect not only the productivity of agriculture but also natural ecosystems to the point of jeopardizing the viability of plants [1,2]. Anthropogenic CO_2_ accumulation and other greenhouse gases in the atmosphere have caused around 100% of the warming observed since 1950 according to the Intergovernmental Panel on Climate Change (IPPC) [3]. In addition, the projected increased frequency of extreme weather events, including devastating droughts, hurricanes, ice storms, and heat waves, is expected to reduce agricultural productivity. A recently published meta-analysis found that future food demand will increase by 35–56% over the period 2010–2050 [4]. With the backdrop of a constantly growing world population combined with production losses due to climate change, securing the food supply and mitigating the effects of global warming are becoming the most urgent challenges in agriculture. Thus, to ensure world food security, there is an urgent need to find sustainable solutions to improve adaptability and resilience of plants to changing climatic conditions without crop yield losses [5]. Although advances have been made to gain insights into the regulatory mechanisms underlying plants’ response to abiotic stress adaptation and tolerance, we are still far from fully understanding them [6,7].

Due to their sessile nature, plants exhibit rapid perception of abiotic stress signals and activate a robust signal transduction machinery, resulting in a stress-specific response signature [8]. Abiotic stresses (including drought, salt, temperature, and flooding) are major causes hampering agricultural productivity by disturbing plant growth through overproduction of reactive oxygen species (ROS) [2,9]. The primary function of ROS (e.g., O_2_^−^, H_2_O_2_, OH˙, ^1^O_2_) is to act as key signal transduction molecules, regulating different pathways during plant stress acclimation. However, they are also toxic by-products that cause damage to proteins, lipids, carbohydrates and DNA, finally leading to interruption of cellular homeostasis and consequently to cell death. Therefore, to adjust growth and productivity under stressful conditions, plants need (i) ROS to adjust their metabolism and build a proper acclimatization response and (ii) sufficient reserves to detoxify ROS [10]. Aside from ROS, abiotic stresses produce numerous other early plant stress responses, such as changes in pH and extracellular ATP (eATP) levels, and stimulation by Ca^2+^ influx via channels. These non-specific passive responses integrate activation of downstream stress-specific hormonal signaling pathways, which can be referred to as active stress responses. Direct or indirect activation of stress-specific transcription factors and genes by plant hormones activates a complex network of defense mechanisms, including biosynthesis of secondary metabolites [11].

Plant hormones, also known as phytohormones, are signaling molecules produced at low concentrations that regulate plant growth and development under both optimal and stress conditions. Among the nine well-characterized plant hormones, ethylene and JAs are typically considered stress hormones, along with abscisic acid (ABA) and salicylates (SAs). Other hormones, including auxins, cytokinins (CKs), gibberellins (GAs), brassinosteroids (BRs), and strigolactones (SLs), are classified as growth-promoting hormones [12]. Plant hormones, as previously thought, do not act alone but in complex networks, and, here, JAs and ethylene present a fascinating case of synergism and antagonism. While they are generally considered as defense hormones that act synergistically against pathogen attacks, they act antagonistically, for instance, in promoting the apical hook of etiolated seedlings, wounding responses, or ozone stress [13,14]. However, we are still far from fully understanding the complex ethylene/JAs interaction network under abiotic stress conditions.

Secondary metabolites are considered derivatives of primary metabolites to enhance plant growth and survival of a plant under various environmental stresses. For instance, they effectively minimize harmful effects of ROS [11]. Conventional breeding programs focused primarily on improving crop productivity. As a result, domestication of crop plants has led to alteration and even loss in diversity of secondary metabolites, making them significantly more susceptible to environmental stress conditions over time [15]. To still ensure high yield, use of, e.g., irrigation, herbicides, insecticides, etc., has become increasingly necessary. However, these agricultural practices are unsustainable and urgently require re-evaluation towards a sustainable and resilient crop production [16]. Future plant breeding programs and metabolite engineering that focus on the beneficial properties of plant hormones and secondary metabolites for climate change adaptation could help to sustainably balance crop yields and biomass losses.

This review seeks to summarize the current information on the impact of ethylene and JAs on secondary metabolites under abiotic stress conditions. We will provide an overview of molecular signaling of ethylene and JAs occurring under different abiotic stresses and what is known so far about their interaction. We will describe the effects of secondary metabolites on plant tolerance and adaptation and the regulatory effects of ethylene and JAs on them. Understanding perception, signaling, and plant responses is crucial to achieve tolerant plants to abiotic stress, with plant hormones and secondary metabolites likely to play key roles. We will mainly focus on the different abiotic stress conditions that are more likely to occur under climate change.

## 2. Ethylene and Jasmonates

While ethylene is the simplest known olefin, JAs include its free acid and a number of conjugates. Both ethylene and JAs occur in almost all tissues of higher plants [17,18]. 

### 2.1. Ethylene Biosynthesis and Signaling 

The biosynthetic and signaling pathways of ethylene have been excellently reviewed, such as in Johnson and Ecker [19] or Pattyn et al. [20]. Briefly, the ethylene biosynthetic pathway consists of three enzymatic reaction steps. In the first step, enzyme *S*-adenosylmethionine (SAM) synthetase converts methionine into *S*-adenosyl-methionine (*S*-AdoMet). In the following, enzyme 1-aminocyclopropane-1-carboxylate (ACC) synthase (ACS) converts *S*-AdoMet directly into ethylene precursor ACC. Finally, ACC oxidation leads to formation of ethylene via enzyme ACC oxidase (ACO), which requires oxygen as a co-substrate and activator. Meanwhile, 5′-methylthioadenosine (MTA) is formed as a by-product of ACC synthesis and is then recycled to methionine via the Yang cycle. This maintains a methionine pool even when ethylene is being rapidly synthesized [21,22]. Most studies have focused on characterizing ACSs as key enzymes since they have been considered the rate-limiting step in ethylene synthesis [23]. For instance, the *Arabidopsis* genome encodes nine *ACS* genes, while *ACS2*, *ACS4-9*, and *ACS11* encode functional ACS; *ACS1* encodes catalytically inactive enzymes or non-functional homodimers [24]. However, over the years, increasing numbers of studies have shown that, in certain specific processes of ethylene biosynthesis, ACO is the rate-limiting step [25], such as during flooding of tomato and *Rumex palustris* [26,27]. In rice, it was found that *ACO* genes *ACO8* and *ACO3* are strongly induced in rice shoots during flooding, while *ACO1* is negatively regulated [28]. Although the biosynthetic pathway of ethylene is straightforward compared to other plant hormones, its production is tightly controlled at multiple levels to ensure optimized developmental and stress-induced ethylene synthesis [29,30,31,32].

**Figure 1:**
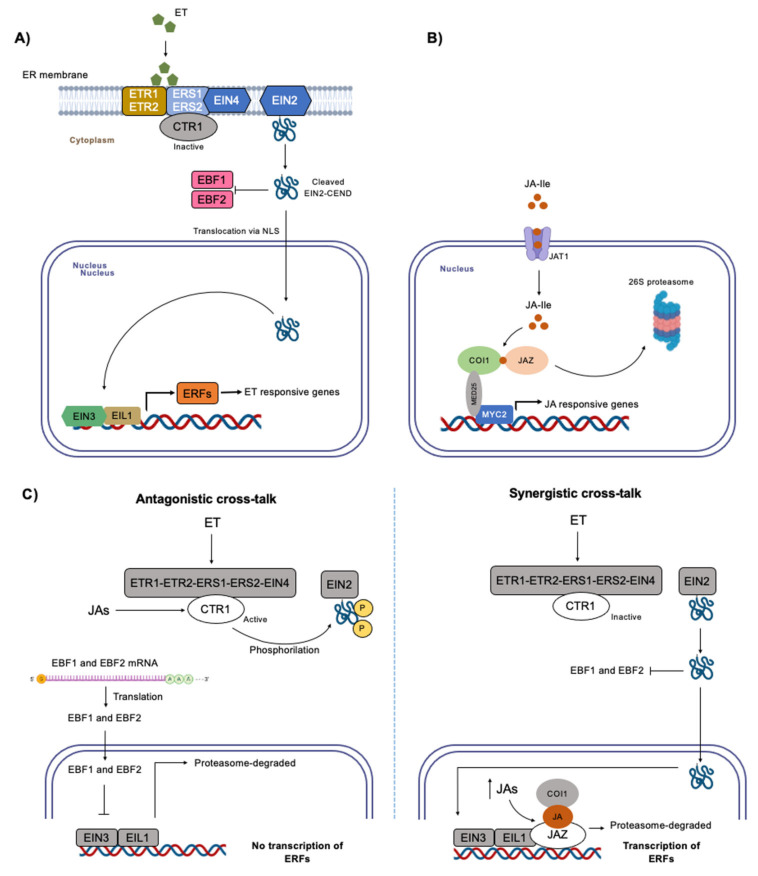
Ethylene and JAs signaling. (**A**) Ethylene signaling. In the presence of ethylene, receptors ETR1, ETR2, ERS1, ERS2, and EIN4 perceive ethylene and deactivate CTR1. EIN2-CEND is cleaved and released from EIN2 and inhibits translation of inhibitors EBF1 and EBF2. EIN2-CEND translocates to the nucleus and induces EIN3/EIL1, which in turn activates ERFs, and, finally, ERFs induce transcription of ethylene responsive genes. (**B**) JAs signaling. When JA-Ile accumulates in the cytosol, it enters the nucleus via JAT1. Then, it binds to protein COI1 from the SCF^COI1^ complex, and, later, they recruit JAZ to form a temporary complex to promote degradation of JAZ. Upon this degradation, MYC2 that is interacting with MED25 is liberated and induces transcription of JA-responsive genes. (**C**) Proposed signaling pathway for ethylene and JAs cross-talk under abiotic stress. Two types of cross-talk have been described for ethylene and JAs. In an antagonistic cross-talk, JAs reactivate CTR1 after ET perception in the cytosol. CTR1 phosphorylates EIN2-CEND, inactivating it. In this manner, translation of EBF1 and EBF2 mRNA takes place and EBF1 and EBF2 enter the nucleus to repress EIN3/EIL1 by promoting its degradation. The result is no transcription of ERFs. In a synergistic cross-talk, both ethylene and JAs promote transcription of ERFs. ET does it through its usual pathway. The increase in JAs in the nucleus promotes formation of the COI1–JA–JAZ complex. JAZ repressing EIN3/EIL1 is degraded, and, hence, transcription of ERFs takes place. ET, ethylene; ETR1/2, Ethylene response 1/2; ERS1/2, Ethylene reticulum sensor 1/2; EIN4/2/3, Ethylene insensitive 4/2/3; CTR1, Constitutive triple response 1; EBF1/2, Ethylene insensitive3-Binding F-Box protein 1/2; EIN2-CEND, C-Terminal end of EIN2; EIL1, Ethylene Insensitive-Like Protein 1; ERFs, Ethylene transcription factors; JA-Ile, jasmonate-isoleucine; JAT1, jasmonate transporter 1; COI, CORANATINE INSENSITIVE 1; SCF, SCF E3 ubiquitin ligase complex; JAZ, JAZ ZIMDOMAIN; MYC2, basic- helix-loop-helix (bHLH) transcription factor; MED25, Mediator subunit 25. Part of this figure was created with Biorender.com (accessed on 20 February 2023).

Ethylene signaling includes the following main components: five ethylene receptors (Ethylene Response 1 (ETR1), ETR2, Ethylene Reticulum Sensor 1 (ERS1), ERS2, and Ethylene Insensitive 4 (EIN4)), a negative regulator Constitutive Triple Response 1 (CTR1), an ER-localized membrane protein EIN2, Ethylene Insensitive3-Binding F-Box Protein1 (EBF1) and EBF2, primary transcription factors EIN3/Ethylene Insensitive-Like Protein1 (EIL1), and ethylene response factors (ERFs) [33] (Figure 1A). As a gaseous plant hormone, ethylene appears to be able to diffuse freely across plant and plasma membranes until it binds to ethylene receptors anchored in the ER membrane to stimulate ethylene responses [34]. In the absence of ethylene, CTR1 is activated by the ethylene receptors and subsequently turns off EIN2 through phosphorylation of its C-terminal end (EIN2-CEND). Finally, involving F-box proteins EBF1 and EBF2, EIN3/EIL1 are degraded in the nucleus, preventing ethylene responses [35,36]. In the presence of ethylene, its binding to the receptors leads to inactivation of CTR1, while EIN2 is dephosphorylated and cleaved. The released EIN2-CEND represses translation of EB1 and EBF2 transcripts in the cytosol and subsequently enters the nucleus, where it directly or indirectly promotes activity of EIN3/EIL1 (Figure 1A) [36,37]. Thus, a transcriptional cascade is initiated, leading to activation and repression of hundreds of ethylene-responsive target genes, such as ERFs [38]. 

### 2.2. Jasmonates Biosynthesis and Signaling

JAs owe their name to *Jasminum grandiflorum*, where they were first discovered [39], but it was not until after several years that their functions in plants began to be elucidated [40,41]. Among the most active JAs, we find jasmonic acid (JA), methyl-jasmonate (MeJA), jasmonate-isoleucine (JA-Ile) [42,43], and 12-oxo-phytodienoic acid (OPDA) [44]. 

JAs biosynthesis has been extensively studied and is well-reviewed [45]. OPDA is the precursor of JAs and is formed in the chloroplast from the polyunsaturated fatty acid α-linolenic, which is released from membrane lipids [46]. This is the start of the 18:3 biosynthetic pathway and is catalyzed by lipases, such as 13-LOX (13-lipoxygenase). Then, allene oxide synthase (AOS) and allene oxide cyclase (AOC) yield OPDA through dehydration-cyclization. OPDA is transported to the peroxisome via the transporter JASSY [47] and partially by CTS ABC transporter (ATP-binding cassette COMATOSE) [48]. OPDA is reduced to OPC-8:0 (8-((1S,2S)-3-oxo-2-((Z)-pent-2-en-1-yl)cyclopentyl)octanoic acid) by OPDA reductase 3 (OPR3) and then activated to OPC-8-CoA. After three rounds of β-oxidation, the final compound obtained in the peroxisome is (+)-7-iso-JA, which is released into the cytosol and can be catalyzed into jasmonate-isoleucine (JA-Ile) by JAR1 (Jasmonate-amido synthetase 1), which will be involved in JA signaling acting on gene expression. Other derivatives of JA can also be formed in the cytosol, such as methyl-jasmonate (MeJA), glycosylated forms, and conjugated forms with amino acids, among others [49]. Recently, an alternative pathway of JA biosynthesis has been postulated and is independent of OPR3 [50]. Instead, once OPDA enters the peroxisome, it is β-oxidized to dnOPDA (2,3-Dinor-12-oxo-10,15(Z)-phytodienoic acid), tnOPDA (deuterated tetranor-OPDA), and finally to 4, 5-ddh-JA (4, 5-didehydro-JA). The latter is transported to the cytosol and reduced to JA by OPR2, an additional OPR enzyme. OPR3 is not present either in liverworts nor mosses [51,52], suggesting that the OPR3-independent pathway is more ancient in the plant lineage and that the OPR3 pathway is preferred in vascular plants [53].

JA-Ile is the most biologically active of the JAs, being crucial in JA signaling (Figure 1B). When JA-Ile levels increase, the conjugate form is transported into the nucleus by JAT1/ABCG16 (ATP binding cassette (ABC) transporter) [54]. Then, JA-Ile binds to the F-Box protein COI1 (CORANATINE INSENSITIVE 1 [55] of the SCF E3 ubiquitin ligase complex (SCF^COI1^), and, later, they recruit JAZ ZIM-DOMAIN (JAZ), forming the temporary ternary complex of COI1–JA–JAZ [56,57]. JAZs, under no-stress conditions, are bonded to MYC TFs through NINJA adaptor protein (JAZ-bound NOVEL INTERACTOR OF JAZ) and recruit TOPLESS scaffolding protein, repressing JA-responsive genes [58]. With formation of the COI1–JA–JAZ complex, JAZs are degraded by the 26S proteasome; MYC2 (the master transcriptional factor), which is interacting with MED25 [59], is liberated and gene expression is induced (Figure 1B) [60,61,62]. In lower plants, JA-Ile is not the ligand to COI1; in its place, dnOPDA from the OPR3-independent pathway binds to COI1 [53] and JAZ proteins possess a single ortholog [63]. Even though the OPR3-independent pathway is characteristic of lower plants, such as liverworts, it is also present in higher plants [50], making OPDA (or its derivatives) also an active form of JAs that can trigger gene expression [64].

## 3. Ethylene and Jasmonates Action and Response Mechanism under Abiotic Stress

Plants are exposed to environmental perturbations throughout their life cycle. Despite these unfavorable circumstances, plants try to adjust their lifestyle by developing a variety of strategies coordinated by plant hormones, resulting in a stress-specific phenotype [65,66]. Both ethylene and JAs regulate developmental and physiological processes (e.g., root development, accumulation of anthocyanins) in a complex manner. Here, we are systematically updating the current information on the impact of major abiotic stresses in the context of climate change, including drought, salt, temperature, and flooding.

### 3.1. Ethylene 

As climate change progresses, droughts are expected to become more frequent, severe, and pervasive. In general, drought stress is characterized by inadequacy of water, which restricts a plant’s genetically determined yield [67]. Ethylene has been associated as a major player in growth inhibition, with ACC appearing to act as a long-distance signal from root to shoot [68]. The mechanism underlying stomatal control is of crucial interest as it controls transpiration and water loss and critically affects drought tolerance of plants. Ethylene has been reported to be involved in both stomatal opening and closing [12,17], suggesting that stress severity might play a key role in ethylene’s action and response mechanism. Several studies have observed that ethylene inhibits ABA-induced stomatal closure by influencing *S*-type anion channels and ROS production [69]. Moreover, ethylene receptors might be crucial for stomatal movement. For instance, in the absence of ethylene, ETR1 mediates H_2_O_2_ signaling in the ABA signaling pathway, but, in the presence of ethylene and after ethylene receptor binding, ABA-induced stomatal closing appears to be inhibited [70]. It is noteworthy that ABA inhibits ethylene synthesis under severe drought stress conditions [71]. On the other hand, Shi et al. [72] reported that BRs trigger ethylene synthesis in *Arabidopsis* under drought stress, and ethylene in turn induces ROS production and signaling, leading to stomatal closure. In the ethylene signaling pathway, degradation of EBF1 and EBF2 plays an important role in stabilizing EIN3. Recently, E3 ligase (RING) type *SALT- AND DROUGHT-INDUCED RING FINGER 1 (SDIR1)* gene was found to positively regulate ethylene signaling in *Arabidopsis* by fine-tuning temperature fluctuation in an EIN2-independent manner. Thus, enzyme SDIR1 directly targets EBF1/EBF2 for ubiquitination and proteasome-dependent degradation [73]. Numerous studies have shown that SDIR1 and its orthologues are strongly involved in stress responses, including drought stress resistance, promoting ABA signaling [74,75,76]. Whether they might also be involved in drought-stress-induced ethylene signaling remains to be elucidated. In contrast, the regulatory functions of EIN3/EILs in response to abiotic stress have received considerable attention within the research community. Liu et al. [77] showed that, in mulberry, *MnEIL3*, which resembles the expression pattern of *Arabidopsis EIN3* and *EIL1* genes, was up-regulated under both drought and salt stress. Moreover, the authors suggested that ethylene biosynthetic genes *MnACO1, MnACO2, MnACS1*, and *MnACS3,* which contain Primary Ethylene Response Element (PERE) and EIL Conserved Binding Sequence (ECBS) motifs that have been reported to be EIN3-interactive motifs, might be regulated by EIN3/EILs since their expression levels were up-regulated under drought and salt stress. However, further studies are needed on ethylene production triggered by the EIN3/EIL1–*ACO/ACS* feedback loop and its impact on abiotic stress tolerance. In addition, it is now well-established that ERFs regulate molecular responses in response to a range of abiotic stresses, including drought stress, by binding to Dehydration-Responsive (DRE) elements. For instance, AtERF1 binds in the promoters of the *Responsive to Desiccation 29B (RD29B)* and *RD20* genes regulating drought response in *Arabidopsis thaliana* [78]. Numerous drought-stress-induced ERF genes have been identified in several other plant species, including *Cicer arietinum* [79], *Citrus sinensis* [80], *Glycine max* [81] and *Nicotiana tabacum* [82]. 

While ethylene signaling is a crucial contributor to drought tolerance, it appears to affect salt stress responses both positively and negatively. A comprehensive review article on ethylene and salt stress tolerance was recently published, summarizing the findings up to 2021 [83], so only more recent publications are discussed here. A genome-wide transcriptome and proteome analysis in quinoa to examine its ethylene-regulated salt tolerance revealed involvement of three ERFs and promotion of ROS by ethylene-enhanced SOD activity [84]. Furthermore, the authors showed that a variety of transporters (including a high-affinity K^+^ transporter, four nitrate and phosphate transporters, a cation/H^+^ antiporter, a Na^+^/Ca^2+^ exchanger, and an aquaporin) were involved. These results demonstrate the importance of ethylene for quinoa salt tolerance, with its influence on osmotic adaptation and ion homeostasis appearing to play a crucial role in the salt tolerance mechanism. In mustard plants, application of ethephon (an ethylene releaser) increased availability for proline and GSH formation. Furthermore, ethephon in conjunction with split doses of nitrogen and sulfur significantly improved salt stress tolerance, reversing the inhibitory effect on photosynthesis and plant growth [85]. Ethylene–melatonin interaction appears to mediate salt stress tolerance in wheat through up-regulation antioxidants activity and detoxification of ROS [86]. Moreover, ethylene has been shown to be necessary for salt stress tolerance, conferred by *SIWRKY23*, an osmotic-stress-induced gene in tomato [87].

Cold and freezing stress have been observed with increasing but varying frequency, intensity, and duration in agricultural regions worldwide [88]. Freezing tolerance in plants is apparently mediated by ethylene in a species-dependent manner. For instance, in *Arabidopsis,* EIN3 has been found to act as a negative regulator of freezing tolerance through negative regulation of the C-repeat binding factor (CBF) pathway [89]. CBF family transcription factors are key regulators of *Cold-Responsive* (*COR*) genes. However, in apple seedlings, transcription factor MdERF1B was reported to up-regulate *MdCBF1*, resulting in improved cold stress [90]. In addition, ethylene appears to have a positive effect on post-harvest cold tolerance of tomato fruits as ethylene biosynthesis inhibitor 1-methylcyclopropene (1-MCP) reduced tomato cold tolerance [91]. Furthermore, it has been suggested that ethylene levels seem to be a key factor in its positive or negative regulation, with ethylene homeostasis appearing to be crucial for freezing tolerance. RARE COLD INDUCIBLE 1A (RCI1A) has been found to interact with ACC SYNTHASE isoforms to maintain adequate levels of ethylene required to promote *COR* gene expression and, hence, freezing tolerance [92]. 

Heat stress or shock defines a transient increase in ambient temperature of 10–15 °C [93]. Due to global warming, the interest in understanding the regulatory mechanisms of heat stress is increasing significantly. For instance, ethephon pretreatment of developing tomato pollen increased ROS detoxification and expression of proteins involved in protein, carbohydrate, and energy homeostasis. This resulted in maintenance of pollen quality despite heat stress, indicating ethylene-mediated pollen thermotolerance [94]. Virus-induced gene silencing of *LlERF110* in lilies resulted in reduced expression of *LlHsfA2, LlHsfA3A,* and *LlHsfA5* (involved in the heat shock factor (HSF)–heat shock proteins (HSP) signaling pathway), as well as *LlHsp17.6* and *LlHsp22* whichseem to protect proteins under heat stress. Ethylene signaling via LlERF110 appears to play a crucial role in lily basal thermotolerance through regulation of the HSF–HSP signaling pathway [95] Basal thermotolerance targets the plant’s ability to resist heat stress, while acquired thermotolerance or thermopriming is a phenomenon based on improved heat tolerance due to previous exposure to sublethal heat stress [96], of which the following study provides an example. In *Arabidopsis,* ethylene signaling has been proposed to induce heat tolerance by transcriptionally activating ERF95 and ERF97 through EIN3, both of which then activate Heat Shock Factor A2 (HSFA2). Subsequently, hub protein HSFA2 binds downstream to the promoters of *Heat Shock* (HS) genes, such as *Ascorbate Peroxidase 2 (APX2)* and *Heat Shock Protein 18.2 (HSP18.2)*, inducing development of heat tolerance [97,98]. Interestingly, this is associated with accumulation of hypermethylation markers (H3K4me3 and H3K4me2) at the promoter of memory gene loci and allows enhanced expression of HS memory genes upon recurrent stress [96]. Thus, ethylene signaling appears to be involved in acquired thermotolerance via HS memory. However, whether ethylene exclusively mediates HS memory via HSFA2 remains to be elucidated.

The increasing frequency of global flooding is causing serious environmental damage and is associated with significant losses in crop production worldwide. Ethylene is a well-established master regulator in flooding tolerance [99]. Upon complete flooding, ethylene induces the SUBmergence 1A (SUB1A) transcription factor, which regulates desiccation tolerance in aerial tissues during the post-flood recovery phase by limiting ROS signaling [100,101]. Furthermore, SUB1A suppresses GA-mediated stem elongation via activation of SLENDER RICE1 (SLR1) and SLENDER RICE-LIKE 1 (SLRL1) to protect flowering responses and ensure plant survival under complete flooding [102,103]. In contrast, during partial immersion, ethylene signaling induces the escape response through stem elongation by enhancing GAs action and suppressing ABA signaling [104]. Interestingly, the SNORKEL (SK) locus encodes two ERF transcription factors: SK1 and SK2, and expression of these genes induces shoot elongation through activating GA responses even in non-flooded plants [105]. Flooding results in impaired gas diffusion in plants with subsequent O_2_ starvation (hypoxia), which is associated with ethylene entrapment and perception in submerged shoot and root tissues. In rice, ethylene induces hypoxia tolerance by regulating proliferation of constitutive aerenchyma forms during flooding and hypoxia [106]. ERF group VII (ERFVII) transcription factors, including PETALA2 12 (RAP2.12), RAP2.2, hypoxia-responsive1 (HRE1), and HRE2, are proposed as key to hypoxia acclimation by helping to maintain homeostasis during reoxygenation stress and contributing to ROS detoxification [99], while PHYTOGLOBINs (PGBs) and PROTEOLYSIS6 (PRT6) appear to have a direct effect on ethylene-enhanced ROS scavenging capacity [107]. 

In summary, the ethylene response is a target for complex abiotic stress regulation, in which EIN3/EIL1 and ERFs TFs are important regulatory hubs. Moreover, increasing evidence suggests a key role for ethylene concentration and homeostasis in abiotic stress tolerance, including an EIN3/EIL1-activated *ACO/ACS* feedback loop. However, a more detailed characterization is required that also considers the species-dependent ethylene stress response. Furthermore, a detailed analysis of the nucleotide context around EIN3 binding sites also needs to be clarified. Whether an EIN3/EIL1-independent ethylene signaling pathway plays a role in abiotic stress tolerance remains to be elucidated.

### 3.2. Jasmonates

JAs have a clear role in drought tolerance [108,109]. One of the proposed explanations for this role is retrograde signaling from plastids to the nucleus [110]. Drought triggers accumulation of antioxidants to prevent oxidative stress and can cause lipid peroxidation of plastids’ membranes. At the same time, lipid peroxidation can derive in synthesis of oxylipins, including JAs, which implies complex cross-talk between JAs and antioxidants. This relationship was recently confirmed in controlled conditions exogenously applying MeJA to leaf discs of Cistus albidus, which resulted in an increase in the precursor of vitamin E [111]. The antioxidant activity of sugar beet was also enhanced under water deficit with application of JA [112].

JAs, especially MeJA, have a well-established role in stomatal closure [113,114,115] —one of the first responses of plants to water scarcity. More recently, OPDA alone has been proposed as a drought-responsive regulator of stomatal closure that works cooperatively with abscisic acid (ABA) [116,117,118]. In fact, there are increasingly more studies that provide evidence of OPDA being a major player in drought response (see [119]). OPDA levels increased in drought surviving plants of Cistus albidus [120], under low VPD conditions in high-mountain plant Saxifraga longifolia [121], and concomitantly with drying soil in tomato [122]. 

Although the role of OPDA in drought tolerance and resistance is clear, the underlying signaling pathway remains elusive [123]. With the discovery of the JAZ family and, consequently, the mechanism through which JA-Ile is perceived by the SCFCOI1–JAZ co-receptor complex, other JAs were tested as ligands for binding with the complex, including OPDA [124]. These studies concluded that OPDA could not be a ligand for the SCFCOI1–JAZ co-receptor complex and other COI1-independent pathways have been proposed [18]. Some hope arose when the conjugated form OPDA-Ile was discovered [125], although, based on subsequent studies, its role in signaling seems to be only of minor biological relevance [123,126]. Similarly, as discussed before, dnOPDA has a major role in lower plants and its action in vascular plants seems to occur when the principal JAs biosynthesis pathway is hampered [50].

JAs have a well-proven role in alleviating salt stress [127]. They have been reported to directly act in roots and increase the antioxidant response. In the meristematic zone of *Arabidopsis* roots, JAZ transcript levels increased under salt stress, evidencing activation of the COI1-dependent signaling pathway [128]. Similarly, JAZ genes were salt-stress-regulated in leaves and roots of tomato and in roots of cotton [129,130]. In another transcript profile analysis, Zhang and colleagues [131] revealed that JA-related genes, such as lipoxygenase (*LOX*), allene oxide synthase (*AOS*), OPDA reductase 3 (*OPR3*), or *COI1,* were up-regulated in a salt-tolerant sweet potato variety under salt stress. These results agree with a previous study in *Arabidopsis* where a lipoxygenase mutant (*lox3* mutants) showed hypersensitivity to salt stress, and this was alleviated by MeJA exogenous application [132]. MYC2 is also a demonstrated player in activation of JA signaling to salt stress, inhibiting cell elongation and regulating proline biosynthesis [128,133]. MYC2 regulates the gene *RD22* (*Responsive to desiccation 22*), a gene that is inducible both by salt and ABA, which implies a cross-talk between JAs and ABA under salt stress [134].

The antioxidant response is enhanced by JAs in salt-stressed plants. Several enzymatic antioxidants have been proven to be up-regulated with JAs treatment. For instance, the activities of superoxide dismutase (SOD), peroxidase (POD), catalase (CAT), and ascorbate peroxidase (APX) were enhanced with exogenous treatments of JA in wheat under salt stress [135]. In rice, salt stress also triggered accumulation of POD but not that of other enzymatic antioxidants [136], and, in forage sorghum, POD and SOD activities increased but not that of CAT [137]. MeJA alleviated salt stress in *Robinia pseudoacacia* by boosting the activity of SOD and APX [138], while, in strawberry, SOD, POD, and APX activities increased [137]. It is interesting to note that application of JAs did not enhance the activity of CAT in most cases. Recently, a study in *Arabidopsis* seedlings found that JA repressed *CAT2* expression through MYC2 [139], suggesting that the role of JA in salt stress is developmental-stage-dependent. 

Application of exogenous JAs not only enhances activity of enzymatic antioxidants but could also trigger accumulation of lipophilic antioxidants. For instance, Qiu et al. [135] reported an increase in carotenoids in wheat with exogenous JA treatment under salt stress and Sheteiwy et al. [140] an accumulation of α-tocopherol. Moreover, application of JAs can also increase photosynthetic activity. For instance, JAs increased the content of photosynthetic pigments or increased net photosynthesis in vine [141], soybean [142], *Anchusa italica* [143], *Limonium bicolor* [144] and strawberry [137].

Another important function of JAs under salt stress is controlling ion homeostasis, hence reducing toxicity for the plant. JAs treatment has been reported to decrease Na^+^ contents in wheat, rice, maize, and strawberry plants [136,137,140,145]. Sodium ions also decreased in the salt-tolerant plant *Anchusa italica* upon MeJA treatment [143]. Wu and colleagues [131] found that the major player in the mechanism behind ion homeostasis under salt stress in rice is OsJAZ9, which interacts with bHLH transcription factors, including OsbHLH062, and this can promote the transcription of several ion transporter genes. 

JAs are crucial regulators of the cold stress response through the ICE–CBF pathway [146]. While exogenously applied MeJA improved freezing tolerance, JA biosynthesis mutants showed hypersensitivity to freezing stress. Furthermore, overexpression of *JAZ1* or *JAZ4* repressed expression of CBF/DREB1, the regulon that is activated by ICE1 (inducer of CBF expression 1 (ICE1)) during cold acclimation [147], hence blocking the cold stress response. Confirming JAs role in cold tolerance, JA-biosynthesis-related genes *OsDAD1*, *OsLOX2*, *OsAOC*, *OsAOS1*, *OsAOS2*, *OsOPR1,* and *OsOPR7* were up-regulated in cold-treated rice seedlings [148]. Transcripts of JA-related genes also accumulated in the cold-tolerant plant *Camellia japonica* under cold stress [149]. Recently, another JA-mediated cold response regulator has been found in apple [150]. B-box protein BBX37 binds to the ICE1–CBF complex and JAZ degradation occurs, therefore promoting cold tolerance. 

Cold stress is particularly relevant for exported fruits. Fruits such as banana or mango exhibit injuries when stored at cold temperatures—i.e., chilling injury. Application of exogenous MeJA has been observed to confer chilling tolerance. For instance, MeJA led to chilling tolerance in banana through induction of the MYC2a and MYC2b transcription factors and interacting with ICE1. Regarding the signaling mechanism of cold tolerance, Ba et al. discovered a lateral organ boundaries domain (LBD) protein that accumulated upon cold stress in banana and that trans-activated expression of *AOC2* [151]. MeJA also reduced chilling injury in mango, guava, loquat fruits, and peach [152,153,154,155]. Chilling stress was also alleviated by MeJA in cherry tomato [156]. More recently, upon application of MeJA, transcription factor *NAC1* has been reported to be up-regulated in peach after cold storage [157].

Since Clarke and colleagues discovered that MeJA had a positive effect on ameliorating heat stress in *Arabidopsis* [158], several studies on JAs role in thermotolerance have been made. Under high light and heat stress (a combination of stresses common in temperate and subtropic climates), transcripts of JA-related genes were accumulated in *Arabidopsis* [159]. In this study, the authors further confirmed the role of JAs in heat stress by using a JAs-signaling-deficient mutant, which resulted to be more sensitive to stress. Overexpression of LOX13—essential enzyme for JAs biosynthesis—resulted in better tolerance to high temperatures in tomato [160]. In wheat, MeJA triggered the accumulation of protein D1 of the PSII under heat stress, protecting the photosynthetic apparatus and avoiding photoinhibition [161]. Several studies have also demonstrated the positive role of JAs in male sterility due to heat stress. For instance, JA and MeJA allowed spikelet opening in heat-stressed rice [162]. These results agree with those of Chen et al., where MeJA enhanced stigma vitality [163]. Similarly, exogenous JA rescued tomato stigma exertion under heat stress [164].

Heat stress induces synthesis of HSP, which is necessary for thermotolerance. The cofactor SGT1 (SUPPRESSOR OF G2 ALLELE OF SKP1), which acts as a cofactor of HSP90, has been proposed to be involved in JA signaling, stabilizing the COI protein [165]. Moreover, WRKY proteins are also believed to be involved in JA signaling in plants exposed to high temperatures. Particularly, JA induced the accumulation of *WKRY40* transcripts under heat stress in pepper and tobacco, conferring heat stress tolerance [166]. More recently, module CsbZIP2-miR9748-CsNPF4.4 (Basic Leucine Zipper Domain transcription factor 2—miRNA family 9748—NITRATE TRANSPORTER 1/PEPTIDE TRANSPORTER FAMILY 4.4) has been reported to confer high temperature tolerance in cucumber through the JA signaling pathway [167].

While ethylene has a clear role during waterlogging [168,169], JAs involvement is not as clear. In *Arabidopsis,* it was found that contents of JAs increased during the first hours of hypoxia but later decreased [170]. Nevertheless, JAs have been reported to have a more established role in reoxygenation after hypoxia [171]. Reoxygenation is thought to be followed by a peak in lipid peroxidation [172], which in turn activates synthesis of oxylipins, including JAs [173]. Yuan et al. found that application of MeJA resulted in higher tolerance to reoxygenation, while JA biosynthesis mutants were very sensitive to it [171]. Moreover, during reoxygenation, most of the genes related to JA biosynthesis were up-regulated as well as antioxidant-related genes, such as *VTC* genes or *GSH* genes, which encode for ascorbate (vitamin C) and glutathione, respectively. Recently, JA-related transcription factors were up-regulated in a waterlogging-tolerant species (*Vigna radiata*) under short-term waterlogging [174].

In summary, JAs are crucial for the abiotic stress response. They have been reported to have a role in drought tolerance, salt stress, cold and freezing acclimation, thermotolerance, and in reoxygenation after flooding. Numerous proteins, transcription factors, and complexes are involved in JAs response to abiotic stress, making it a complex network that is species- and stress-dependent. Retrograde signaling seems to be fundamental for drought, salt stress, and flooding. Upon these stresses, lipid peroxidation occurs as a result of the increase in ROS, which in turn promotes synthesis of oxylipins, including JAs, and finally induction of antioxidant-related genes that will confer tolerance to the plant. The precursor of JAs, OPDA, has a key role in drought tolerance via different signaling pathways to that of JA-Ile. Under salt stress, MYC2, the master regulator of JA-responsive genes, has been reported to regulate the gene responsive to dehydration 22, induced by ABA upon dehydration [175]. JAs are also involved in cold acclimation by possibly activating the ICE1–CBF complex and by up-regulating NAC TFs, which are identified as cold stress regulators [176]. Finally, JAs are key to avoid male sterility under high temperatures and have been proposed to confer thermotolerance to high temperatures through stabilization of the COI protein by SGT1 but also via interaction with other proteins and cofactors.

### 3.3. Ethylene and Jasmonates Cross-Talk under Abiotic Stress

Ethylene and JAs have essential roles in abiotic stress responses. Ethylene and JAs have a clear cross-talk in the pathogen-related plant response [177], but how they interact under abiotic stressors is hardly studied comparatively. Nevertheless, some authors have provided insight regarding their cross-talk under abiotic stress. For instance, ethylene and JAs had a positive role in alleviating selenite toxicity [13]. Moreover, ethylene and JAs may have an antagonistic role in conferring thermotolerance. While JA-deficient mutants displayed hypersensitivity to heat stress and MeJA application protected against heat stress, ethylene-defective mutant *ein2-1* conferred greater thermotolerance [158]. This fact, however, was discussed as being the result of less cell death induced by ethylene. Alternatively, ethylene conferred basal thermotolerance under more extreme temperatures [178]. Hence, ethylene action in heat stressed-plants seems to be dependent on the intensity of the stress. Agreeing with the role of ethylene in promoting cell death, Tuominen et al. found that ethylene spread cell death while JAs protected the tissues under ozone stress [14], hence resulting in an antagonistic relationship. JA is suggested to inhibit ethylene pathway via targeting the constitutive triple response kinase (CTR1). More recently, ethylene and JA have been reported to interact in the flower and fruit development of *Cucurbita pepo* [179]. This cross-talk was confirmed by using JA- (*lox3a*) and ET- (*aco1a* and *etr2b*) deficient mutants and applying exogenous MeJA. Both mutants were rescued by MeJA and developed correctly.

Cross-talk between ethylene and JAs via EIN3/EIL1 has been found in formation of adventitious roots, a process that is crucial under several abiotic stresses [180]. These findings suggest EIN_3_/EIL_1_ as the link between JA and ethylene signaling, which was already proposed in a previous work, where EIN3/EIL1 induction by JA was found to be JAZ-mediated [181]. ERF has also been proven to be a link between ethylene and JA under abiotic stress. Cheng et al. reported that both ethylene and JA induced ERF1 under salt stress and the combination of both hormones resulted in a synergistic effect on ERF1 [78]. Studying the underlying molecular mechanism of apical hook development, Zhang et al. found that the JA master transcription factor MYC2 represses EIN_3_ function by both promoting its degradation and physically binding to EIN3 and inhibiting it [182]. These findings were confirmed the same year by another study, but it seems that it may be a specific response for plant pathogen attacks [183].

Ethylene and JAs cross-talk occurs under some abiotic stresses, and, under other circumstances, they have been found to act either antagonistically or synergistically (Figure 1C). The key link for this cross-talk seems to be EIN3/EIL1: while in the antagonistic cross-talk JAs reactivate CTR1 after ET perception in the cytosol, resulting in inhibition of EIN3/EIL1, in the synergistic cross-talk, both ethylene and JAs promote transcription of ERFs through induction of EIN3/EIL1. Since some of these studies were conducted under controlled conditions, more research on different abiotic stressors, and their combinations, is necessary to fully elucidate the signaling mechanism of the interaction of ethylene and JA and when antagonistic or synergistic cross-talk is occurring.

## 4. Role of Secondary Metabolites under Abiotic Stress 

**Figure 2:**
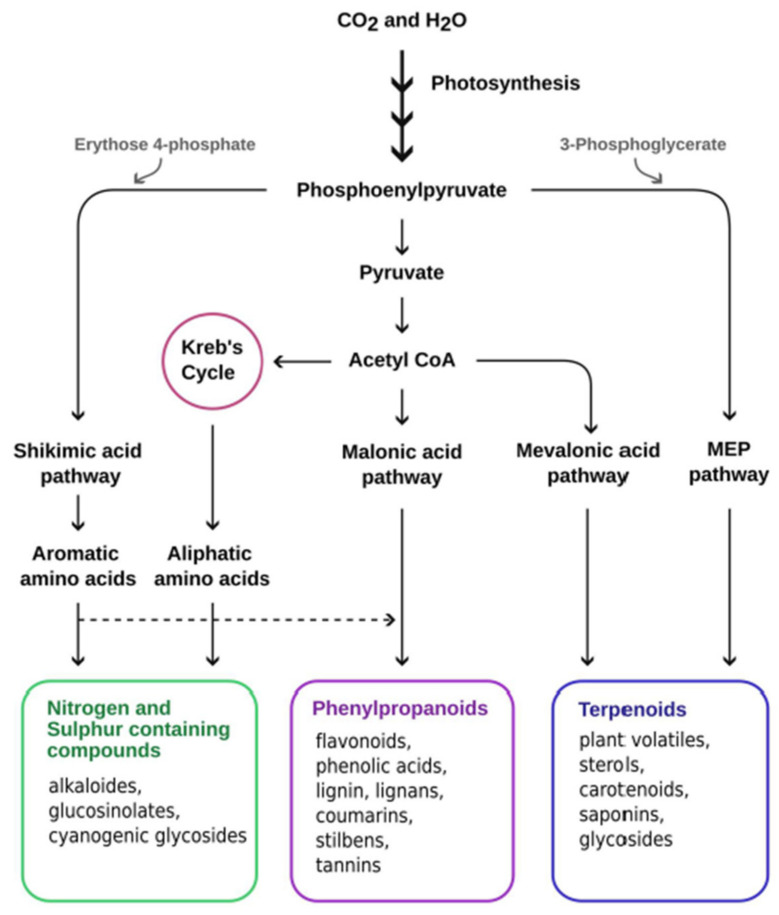
Schematic representation of biosynthesis of secondary metabolites (classified according to their chemical nature) required for acclimation under abiotic stress conditions.

Secondary metabolites are multifunctional organic compounds that play crucial roles in stress acclimatization of plants. Traditionally, they are defined as mediating exclusively plant–environment interactions as opposed to primary metabolites that are directly required for plant growth. Due to improved genetic and analytical techniques, the boundaries between primary and secondary metabolism are becoming increasingly blurred, and secondary metabolites are instead viewed as integrated components of metabolic networks [184]. Based on their chemical nature, they can be classified into (i) terpenoids (plant volatiles, sterols, carotenoids, saponins, and glycosides), (ii) phenolic compounds (flavonoids, phenolic acids, lignin, lignans, coumarins, stilbenes, and tannins), and (iii) nitrogen–sulfur containing compounds (alkaloids, glucosinolates, and cyanogenic glycosides) [185]. A simplified scheme of the primary and intermediate metabolites and pathways, including shikimate, malonate, mevalonate, and ethyl erythritol phosphate, is shown in Figure 2. Plants that have a high level of plasticity in their secondary metabolism, which enables them to generate chemical diversity to an almost unlimited extent through structural and chemical modifications [186,187], are likely to have a selective and adaptive advantage, particularly in the face of climate change challenges.

### 4.1. Secondary Metabolites under Abiotic Stress

Abiotic stresses affect biosynthesis of plant secondary metabolites, which are produced at low concentrations in living plant cells. For instance, heat stress caused an increase in flavonoids and phenolic compounds in *Lens culinaris* [188], terpenoids in *Daucus carota* [189], and alkaloids in *Camptotheca acuminata* [190]. Anthocyanin accumulation has been linked to increased drought tolerance, while in *Hypericum brasiliense* abiotic stress induced the synthesis of secondary metabolites, including rutin, quercetin, and betulinic acid [191]. In *Populus tremula*, changes in lignin content have been associated with cold stress [192]. Moreover, it has been reported that cyanogenic glycosides and glucosinolates respond to climatic stresses, such as drought and elevated temperatures [193]. In maize roots, accumulation of phytoalexins showed increased drought tolerance [194]. Here, we discuss secondary metabolites, including flavonoids and polyphenols, lignin, terpenoids, cyanogenic glucosides, amino acids and derivatives, as well as phytoalexin and glucosinolates, and their impact on abiotic-stressed plants.

### 4.2. Flavonoids and Polyphenols

More than 8000 phenolic compounds have been identified in plants, half of which are flavonoids [195]. Key biosynthesis genes of flavonoid and phenolic compounds such as *Phenylalanine Ammonia Lyase (PAL), Cinnamate 4-Hydroxylase (C4H), 4-Coumarate: CoA ligase (4CL), Chalcone Synthase (CHS), Chalcone Isomerase (CHI), Flavanone 3-Hydroxylase (F3H), Flavonoid 3′-Hydroxylase (F3′H), Flavonoid 3′5′-hydroxylase (F3′5′H), Dihydroflavonol 4-Reductase (DFR), Flavonol synthase (FLS), Isoflavone Synthase (IFS), Isoflavone Reductase (IFR),* and *(UDP Flavonoid Glycosyltransferase (UFGT)* have been found to be up-regulated by abiotic stress [196,197,198,199]. 

*Arabidopsis* transcriptomic and metabolomic studies revealed that enhanced accumulation of flavonoids under drought stress improves resilience [200]; however, the mechanism of action is poorly understood. For instance, increased levels of kaempferol and quercetin improved drought tolerance in tomato plants [201]. In *Amaranthus tricolor* genotype VA3, an increase in phenolic compounds, such as hydroxybenzoic acids, hydroxycinnamic acids, flavonoids, and phenolic acids, was observed under drought stress [202]. Many flavonoids and phenolic compounds act as antioxidants, such as anthocyanins, that catalyze oxygenation reactions to scavenge ROS [200]. In transgenic *Arabidopsis thaliana* plants, overexpression of VvbHLH1 resulted in a significant increase in flavonoid accumulation through regulation of flavonoid biosynthetic pathway genes, conferring in salt tolerance [203]. Similar results could be observed in tobacco, where *NtCHS1*-overexpressing plants showed higher salt tolerance due to increased rutin accumulation and lower H_2_O_2_ and O^−^_2_ levels [204]. The authors revealed that tobacco R2R3 MYB-type repressor NtMYB4 negatively regulates *NtCHS1* expression. Flavone synthase genes *GmFNSII-1* and *GmFNSII-2* were found to be up-regulated under salt stress conditions, leading to increased flavone biosynthesis in *Glycine max* [205]. Increased contents of phenols and flavonoids were also observed in *Hibiscus cannabinus* after salt treatment [206]. Furthermore, plants under temperature stress (both heat and cold) similarly synthesize more phenolic compounds and flavonoids, including anthocyanins, flavonols, and phenolic acids, to protect plants and improve stress tolerance [207,208]. In contrast, results of a recent study on pearl millet testing gene expression of genes involved in the flavonoid biosynthetic pathway, such as *C4H*, *CHS*, *CHI*, *F3′H*, *F3H*, *Shikimate-O-Hydroxycinnamoyltransferase (HCT),* and *Caffeoyl-CoA-O-methyltransferase*, showed that flavonoid synthesis was inhibited under long-term heat stress [209]. The authors suggest that regulation of the flavonoid pathway involves an energy-saving strategy to better withstand high temperatures. Elevated levels of 4-hydroxybenzoic acid, benzoic acid, caffeic acid, coumaric acid, cinnamic acid, gallic acid, homovanillic acid, ferulic acid, salicylic acid, and vanillic acid contributed to increased high-temperature tolerance in *Festuca trachyphylla* plants [210]. Phenylpropanoid biosynthetic transcripts were up-regulated in tartar buckwheat seedlings when exposed to cold stress, which subsequently resulted to accumulation of anthocyanins and proanthocyanidins [211]. A large-scale metabolomics analysis was performed on *Chrysanthemum morifolium* to investigate the influence of flooding on flavonoid synthesis at different growth stages. The authors observed increased flavonoid contents and identified 46 metabolites belonging to the groups of flavone C-glycosides, flavonol, and flavones. Interestingly, however, quercetin, eriodictyol, and most flavone C glycosides were significantly elevated in the stages after flooding stress [212]. In addition, increased levels of flavonoids and phenolic compounds not only help to improve stress adaptation and tolerance but also improve crop quality. Their production is, therefore, also of particular interest for agriculture to increase their contents through targeted cultivation methods. For instance, in four different purple rice varieties, grain anthocyanin content could be increased by a factor of 2 to 5.5 by growing on flooded rather than aerated soils [213].

### 4.3. Lignins

Lignin is a non-linear, heterogeneous biopolymer that accounts for 30% of the organic carbon content in the biosphere [214]. Lignin provides plants with structural rigidity and water impermeability to enhance plant growth and long-distance water transportation. Therefore, lignin is considered as one of the keys of the evolution of terrestrial plants [215]. In fact, early land plants, such as mosses, do not possess lignin, at least in its current form in vascular plants.

Being one of the essential structural components of cell wall, lignin has a key role in plant growth and development. Lignin-deficient mutants are not able to develop correctly and have inhibited growth [216]. Moreover, a mutation in the gene *CH4* (*cinnamate 4-hydroxylase*), which is essential for lignin biosynthesis, results in male sterility [217]. Due to its physical properties, lignin acts as a barrier against pathogens but also acts against abiotic stresses [218]. Lignin functions as a natural casing to avoid water leakage and, therefore, helps in maintaining osmotic equilibrium [219]. Lignin confers drought tolerance and their contents increase under drought. For instance, lignin-biosynthesis-related genes were up-regulated in maize under drought [220,221,222]. Lignin has also been reported to have a role in salt stress [223]. Deposition of lignin induced by SOD contributed to salt tolerance in *Potentilla astrosanguinea* [224]. Lignin has also been found to accumulate along with lignin biosynthetic gene *CH3* in cold-acclimated *Rhododendron* spp. [225]. On the other hand, lignin biosynthesis genes were not up-regulated by cold temperatures in birch [226]. Additionally, lignin biosynthesis has been reported as a response to waterlogging [227].

### 4.4. Terpenoids

Terpenoids or isoprenoids are the largest and most diverse class of chemical compounds in plants [228]. Within the most studied terpenoids (without including phytohormones), we find chlorophylls, carotenoids, and tocochromanols that derive from the MEP pathway (see Figure 2) [229,230,231,232,233].

Under abiotic stress, terpene synthases (TS) are activated to synthesize terpenoids. There are two classical TS and a growing number of novel TS with different structures that perform different reactions [234]. In *Camellia sinensis,* 80 TS-like genes were found, 22 of which had full coding sequences [235]. Upon cold, salt, and drought stress, several TS genes were up-regulated, although many were down-regulated. In a similar analysis, 49 *RcTS*-like genes were found in *Rosa chinensis,* and most of these genes were up-regulated under heat and osmotic stress [236]. Several prenylsynthase-TS were up-regulated under drought and circadian rhythms and under cold stress in the orchid *Dendrobium catenatum* [237]. In seedlings of abiotic-stress-tolerant *Ricinus communis*, 37 of the 46 *RcTS*-like genes were highly responsive to heat stress [238]. 

### 4.5. Cyanogenic Glucosides

Cyanogenic glucosides are a type of specialized bioactive compound with defense functions that are present in ferns, gymnosperms, and angiosperms [239,240]. Their most important function is being effective deterrents for herbivores, but they have also been proposed as carbon and nitrogen transporters and may also have a role in modulating oxidative stress [241]. In high concentrations, cyanogenic glucosides can be fatal to humans and animals. A cyanogenic glucoside that has been extensively studied is dhurrin, which is known for occurring in sorghum at early growth stages [242].

Given cyanogenic glucosides’ accentuated role in biotic stresses, little attention has been paid to abiotic stresses. Nevertheless, some studies have evaluated accumulation of cyanogenic glucosides in sorghum and cassava (another crop that contains cyanogenic glucosides) under abiotic factors. For instance, severe water stress incremented the contents of dhurrin in sorghum leaves [243,244]. With dhurrin contents increasing under drought and sorghum being drought-tolerant, it could seem that cyanogenic glucosides have a role in drought tolerance. However, Sohail and colleagues have discarded this hypothesis by demonstrating that dhurrin has no role in reducing oxidative stress [245]. Dhurrin can serve as a nitrogen remobilizer, which is positive under drought since the activity of nitrate reductase activity is reduced [246]. In cassava, drought and high temperatures increased the cyanide potential (total cyanide released from cyanogenic glucosides) of tubers in a greenhouse experiment [247]. In another experiment testing salt tolerance in cassava, it was found that, under salt treatment, cyanide glucosides increased in leaves in young plants but not in tubers, with no significant changes in older plants either [248]. Even though these studies are not conclusive on the role of cyanide glucosides under abiotic stress, they are important in terms of food security decisions.

### 4.6. Amino Acids and Their Derivatives

Abiotic stress induces free non-protein amino acids that act primarily as osmoprotectants and antioxidants, such as γ-aminobutyric acid (GABA), β-alanine, β-aminobutyric acid, ornithine, and citrulline [249]. For instance, accumulation of citrulline in watermelon has been linked to oxidative stress tolerance during drought [250]. A recent study showed that β-aminobutyric acid acts as an osmoprotectant in *Vica faba* by enriching proline and soluble sugars, which could improve osmotic adaptability under drought stress. Moreover, accumulation of drought-tolerance-related genes (such as *VfMYB*, *VfERF*, *VfNCED*, *VfWRKY*, and *VfHSP*) in leaves and roots after treatment of GABA suggests that it might act as a signaling molecule to regulate expression of these genes [251]. GABA-depleted mutant *gad1/2* showed increased sensitivity to dryness due to a defect in stomatal closure. However, a functional complementation that increased GABA levels reversed the drought-sensitive phenotype of *gad1/2*. Thus, the study revealed that GABA accumulation under drought stress appears to be involved in stomatal regulation [252]. Exogenous application of GABA in mulberry resulted in increased antioxidant enzyme activity and decreased oxygen-induced injury associated with increased salt resistance [253]. In addition, GABA appears to act as a signaling molecule in flooding stress by activating H_2_O_2_ signaling and preventing cells from entering programmed cell death (PCD) [254].

### 4.7. Phytoalexins and Glucosinolates

Phytoalexins are antimicrobial secondary metabolites that are produced by plants as a response to biotic and abiotic stressors [255]. They are synthesized de novo and rapidly increase upon pathogen attack and are present in several families of angiosperms. They are represented by many different chemical classes. For instance, we find biphenyls and dibenzofurans in Rosaceae; hydroxystilbenes in Vitaceae; polyacetylenes in Compositae and Umbelliferae; or tryptophan-derived secondary metabolites in Brassicaceae. See [256] for a complete list of phytoalexins and their presence in different families.

Despite phytoalexins being induced primarily upon pathogen attack, they have also been reported to have a role in response to abiotic stress [257]. For instance, zealexins and kauralexins (acidic terpenoid phytoalexins) accumulated under drought in maize roots and a mutant deficient in kauralexin resulted in hypersensitivity to drought [194]. In grapevine, two phytoalexins (trans-resveratrol and trans-*ε*-viniferin) increased upon ABA treatment but decreased with osmotic stress [258]. Moreover, pretreatment with polyamines (with essential roles in growth and cell proliferation) lowered the defense response of grapevine to *Botrytis cinerea*. These results first suggest that these phytoalexins could have a role in other abiotic stresses other than osmotic stress given its increase after ABA treatment and, second, they confirm the trade-off between growth and defense. Oxidative stress caused accumulation of camalexin in *Arabidopsis* [259], and heavy metal stress imposed by exogenous application of CuCl_2_ elicited accumulation of spirobrassinin and cyclobrassinin in canola [260].

Glucosinolates (GSLs) are also secondary metabolites specialized in plant defense [261]. There are three classes of GSLs: (i) indole glucosinolates, derived from tryptophan; (ii) aliphatic glucosinolates, derived from methionine; and (iii) benzyl glucosinolates, derived from phenylalanine or tyrosine. Depending on the herbivore attack, one group of GSL or other will act [262,263,264]. Unlike cyanogenic glucosides, GSLs are only present in angiosperms, and a family where they are especially diversified is Brassicaceae [265,266]. Interestingly, in the case of *Brassica* spp., indole GSL produces a diverse array of phytoalexins, which have already been mentioned before. 

GSLs not only have a clear role in biotic stress [267] but their contents also fluctuate under abiotic stress. They have been reported to vary under salt and drought stress, extreme temperatures, light stress, and nutrient deficiency [265,268,269]. Their mechanism of action is still not clear, but some light has been shed in recent literature. For instance, Salehin et al. found that auxin-sensitive Aux/IAA repressors IAA5, IAA6, and IAA19 regulated accumulation of GSLs under drought in *Arabidopsis* [270]. Using a *IAA5/6/19*-defective mutant resulted in reduced accumulation of GSLs and malfunctioning of stomatal regulation. Application of exogenous GSLs restored stomatal regulation. On the other hand, an almost-GSL-defective mutant (*myb28myb29*) showed hypersensitivity to ammonium but in a GSL-independent manner [271], which contrasts with another study from the same group where they found that GSL metabolism was stimulated by ammonium, particularly the genes *CYP79F1* and *CYP79F2* from the aliphatic glucosinolate pathway [272]. More recently, the RING finger-containing E3 ubiquitin ligase HIGH EXPRESSION OF OSMOTICALLY RESPONSIVE GENES 1 (HOS1), involved in the cold signaling response, has been proposed as a regulator of GSLs since a *hos1* mutant displayed reduced accumulation of GSLs [273]. 

Thus, it is evident that secondary metabolites play a crucial role in adapting plants to the changing environment and overcoming stress constraints. Furthermore, plants that exhibit high plasticity in their secondary metabolism, allowing them to generate near-infinite chemical diversity through structural and chemical modifications, are likely to have a selective and adaptive advantage, especially in the face of climate change challenges. Therefore, further future studies are needed to elucidate accumulation and quantification of as yet unknown metabolites. Manipulation of selective metabolites, which play crucial roles under abiotic stress, promises to improve crop yield under stressed conditions, which could render herbicide use obsolete. Technologies such as metabolomics and transcriptomics could help fill the gap in our knowledge of hormonal regulatory mechanisms to improve their synthesis.

## 5. Ethylene and Jasmonates Role on Secondary Metabolites under Abiotic Stress

Plant-stress-responsive pathways, regulated by phytohormones, involve biosynthesis of secondary metabolites. Here, we discuss current evidence on the role of ethylene and JAs in synthesis and accumulation of secondary metabolites.

### 5.1. Ethylene and Secondary Metabolites

The ethylene-mediated regulatory network also involves synthesis of secondary metabolites under abiotic stress conditions. Recently, a genome-wide transcriptomic and proteomic study of molecular regulations related to the impact of ethylene on salt tolerance was performed in quinoa, which is known to exhibit pronounced salt tolerance. Numerous proteins associated with secondary metabolism have been shown to be activated in response to ethylene and salt stress in quinoa, such as 3 *CHSs* or *CqCYP76AD1*. CHS catalyzes the first committed step in the flavonoid biosynthetic pathway, while CqCYP76AD1 is involved in biosynthesis of betalains, a group of tyrosine-derived, red–violet, and yellow pigments [84]. Overexpression of *OsERF71* in rice roots under drought conditions led to combinatory overexpression of cell-wall-associated genes, such as lignin biosynthetic genes, thereby inducing greater aerenchyma and radial root growth in rice plants, contributing to drought tolerance [274]. Watkins et al. [275] showed that accumulation of flavonol in guard cell was regulated by ethylene, suggesting that flavonol, in its function as an antioxidant, negatively regulates stomatal closure by ROS scavenging under stressful conditions. Aside from abiotic stress conditions, ethylene-mediated regulation of secondary metabolites, including terpenoids, phenolic compounds, and nitrogenous compounds, has also been demonstrated under biotic stress conditions, fruit ripening, or in vitro experiments [276,277,278,279]. However, we are still far from understanding the complex network of ethylene regulation involved in biosynthesis and accumulation of secondary metabolites that enables plants to rapidly adapt to abiotic stress conditions, overcome negative effects, and gain stress tolerance. Thus, further research is needed to elucidate the role of the ethylene–secondary metabolite complex in the response to abiotic stresses at a detailed biochemical level and provide targets for development of crops that are more resilient to climate change challenges.

### 5.2. Jasmonates and Secondary Metabolites

JAs have a decisive role in abiotic stress response. JAs have been reported to have a major role in enhancing secondary metabolites under abiotic stress [272]. Exogenous MeJA increased the expression of terpene synthases genes in *D. catenatum* and *C. sinensis* [235,237]. In a drought-tolerant pearl millet variety, MeJA also increased expression of terpene synthase genes [280]. Overexpression of *SmAOC*, crucial for JAs biosynthesis, resulted in an increased accumulation of diterpenes and phenolic acids in *Salvia miltiorrhiza*, an important medicinal plant [281]. Genes related to the biosynthesis of the sterol precursor squalene were up-regulated by MeJA in *Panax ginseng* [282]. MeJA has also been reported to increase thr expression of genes of the phenylpropanoid pathway under arsenic stress [283]. Other secondary metabolites have been found to be up-regulated with application of MeJA, namely saponin, anthraquinone, and lignin biosynthetic pathways in *Aloe vera* [284] and glycosidic isoflavones in soybean cell cultures [285]. A mechanism of action of JAs in regulating secondary metabolite rutin has been found in *Fagopyrum tataricum* [286]. JAs-responsive MYB transcription factors repress rutin biosynthesis via repressing phenylalanine ammonia lyase (a key enzyme in the phenylpropanoid pathway), but accumulation of JAs and subsequent degradation of FtJAZ and FtMYBs lead to transcription and rutin biosynthesis.

## 6. Conclusions and Future Perspectives

**Figure 3:**
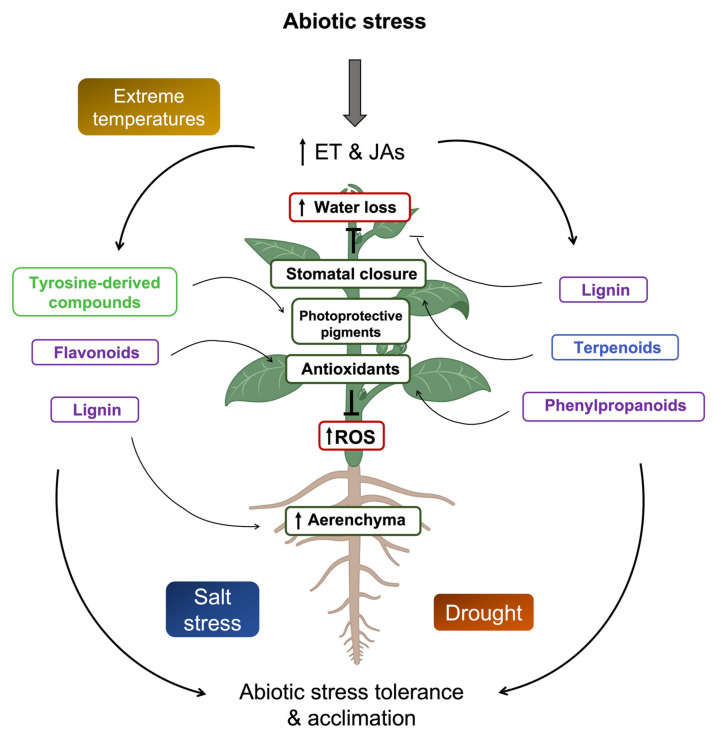
Schematic representation of ethylene and jasmonates effects on secondary metabolites under abiotic stress in plants. Extreme temperatures, salt stress, or drought trigger accumulation of ROS, which can lead to cell damage. Concomitantly, ethylene, JAs, and secondary metabolites are synthesized. Ethylene has been reported to affect accumulation of tyrosine-derived compounds, flavonoids, or lignin. JAs have been shown to trigger synthesis of lignin, terpenoids, or phenylpropanoids. Lignin directly acts in avoiding water loss due to its physical properties and also promotes formation of aerenchyma. Flavonoids and phenylpropanoids, which include numerous antioxidants, scavenge ROS. Terpenoids have a clear role in promoting stomatal closure and, hence, avoiding water loss. Finally, tyrosine-derived compounds, such as betalains—i.e., photoprotective pigments—avoid photoinhibition. All these responses lead to abiotic stress tolerance and acclimation. ET, ethylene; JAs, jasmonates; ROS, reactive oxygen species. Part of this figure was created with Biorender.com (accessed on 9 March 2023).

Given their sessile nature, plants need to acclimate and adapt to heterogenous habitats. Plants have developed a myriad of mechanisms to tolerate and be resilient to abiotic stress. Among these, we find phytohormones and secondary metabolites. It is clear that both ethylene and JAs have a role under abiotic stress and that plants are able to acclimate thanks to their action either alone or in cross-talk. The key links for their interaction under abiotic stresses seem to be EIN3/EIL1 and JAZ-MYC2, which act as nodes connecting multiple signaling and biosynthetic pathways, including that of secondary metabolites. Ethylene and JAs have been reported to have an effect on some secondary metabolites, resulting in increased tolerance to abiotic stress (Figure 3). However, the exact mechanism through which they act remains elusive. More extreme and unpredictable weather conditions caused by climate change press for more research on ethylene, JAs and secondary metabolites in order to define thresholds in plant performance and designate candidate compounds for improving crop resilience.

## Data Availability

Data sharing is not applicable. No new data were generated or analyzed in this study.
